# “It’s so scary, and you’re so alone with it”: Clinicians’ perspectives on suicide risk management in integrated primary care

**DOI:** 10.1371/journal.pmen.0000029

**Published:** 2024-06-04

**Authors:** Celine Larkin, Catarina Kiefe, Esther Boama-Nyarko, Catherine Dube, Aishwarya Khanna, Stephen Erban, Rachel Davis-Martin, Edwin D. Boudreaux

**Affiliations:** 1 Department of Emergency Medicine, UMass Chan Medical School, Worcester, Massachusetts, United States of America; 2 Department of Psychiatry, UMass Chan Medical School, Worcester, Massachusetts, United States of America; 3 Department of Population and Quantitative Health Sciences, UMass Chan Medical School, Worcester, Massachusetts, United States of America; 4 Department of Internal Medicine, UMass Chan Medical School, Worcester, Massachusetts, United States of America; 5 Department of Family Medicine, UMass Chan Medical School, Worcester, Massachusetts, United States of America; University of Luxembourg: Universite du Luxembourg, LUXEMBOURG

## Abstract

Primary care clinics serve many patients experiencing latent or evident suicide risk and may benefit from implementing suicide care improvements such as the Zero Suicide model. However, little is known about the readiness of clinics to implement such initiatives. We interviewed a range of clinicians (e.g., medical providers, behavioral health providers, nurses; n = 24) from six integrated primary care clinics to better understand strengths and limitations of the milieu, how suicide risk is currently detected and managed, and which implementation strategies could be employed to improve suicide prevention. We found clinics were extremely busy and resource-constrained but had a strong and longitudinal commitment to patients and families. Suicide risk was detected in a variety of ways and clinicians had limited resources to offer these patients. Clinicians sought to preserve patients’ autonomy and trust while also ensuring their safety. Preferred strategies included dissemination of protocols and tools, training, electronic health record changes, and improved staffing. Our findings suggest that suicide prevention initiatives in primary care should attend to the constraints of the care setting, adapting their approach to ensure they fit with workflow while also centering patient autonomy and rapport.

## Introduction

There are over 700,000 deaths by suicide globally each year, such that one in every 100 deaths is a suicide [1]. The societal and interpersonal effects of suicide are profound [2,3]. Up to 80% of those who die by suicide presented to a primary care provider in the year before death [4], often for non-psychiatric reasons [5]. Primary care clinics may therefore be the first and only health care setting to which many patients with suicide risk present. Although most primary care clinics do not routinely screen for suicide risk [6], research shows a moderate but significant prevalence of current ideation and past suicide attempts among these patients [7–10]. There are several evidence-based interventions to address suicide risk in outpatient settings, including cognitive-behavioral therapy, dialectical behavior therapy, and the safety planning intervention [11,12].

Previous research indicates varying levels of support for and comfort with suicide prevention efforts, including screening, in diverse healthcare settings. For example, those working in psychiatric settings tend to have more positive attitudes towards patients experiencing suicidal crisis and towards suicide prevention than those working in non-psychiatric settings [13–17]. Surveys of primary care providers have found that almost half had a patient attempt suicide within the past year and most primary care providers screened for suicidality rarely or only when it was indicated [18,19].

Although primary care providers regularly see patients at risk of suicide, extant research suggests that they may not be well-supported or trained to intervene effectively. Training directors of primary care programs report that training around suicide and depression is inadequate [20]. This is important as primary care providers who see themselves as competent at working with patients experiencing suicidal crisis appear to be more willing to assess and treat such patients [21]. In qualitative studies, primary care providers have expressed a lack of confidence in their suicide assessment skills [22–24] and challenges in referring patients to specialty mental health care [25,26].

Suicide prevention interventions can have substantial effects on the risk of suicidal behavior, with outpatient mental health setting-based interventions being especially effective in preventing suicide attempts [27]. There is growing recognition that improving suicide-related care requires a whole-system approach. When applied in health care settings, the Zero Suicide model seeks to better identify, engage, treat, and transition those at risk of suicide by engaging leadership, training, and applying continuous quality improvement (CQI) strategies [28]. There is increasing evidence of this model’s effectiveness in preventing subsequent suicide attempts in mental health settings [29], and of its acceptability to a range of stakeholders from clinicians and administrators [30] to patients and family [31]. However, its fit with primary care is yet to be determined. Although several qualitative studies have examined primary care providers’ perspectives on suicidality [9,25,32,33], they have not examined attitudes specific to components in the Zero Suicide model and seldom include participants from a diverse range of clinical roles. In terms of the "engage” and “treat” components of Zero Suicide, it is unclear what resources and capacity exist in the primary care setting to deliver brief versus more extensive suicide-focused interventions. The current study was a formative evaluation to better understand primary care clinicians’ attitudes towards components in the Zero Suicide model, and was intended to inform the implementation of the model in primary care practices within a large healthcare system [34]. The goal of the study is to present a qualitative analysis of primary care clinicians’ perspectives on suicide risk detection and management.

## Methods

### Setting and participants

The setting for this study was a large healthcare system in Massachusetts, specifically its six facility-based primary care clinics. The clinics had a total of 168,000 visits in 2023, ranging from 16,525 visits for the lowest volume clinic to 46,581 for highest volume clinic. These primary care clinics follow an integrated mental health care model, whereby on-site behavioral health (BH) clinicians collaborate with primary care providers in assessing and intervening with patients experiencing emotional, psychological, social, or psychiatric issues. At the time of this study, each clinic had at least one BH provider (a psychologist or social worker) available for provider consultation, warm hand-offs, joint sessions, or ongoing therapy. Two of the clinics were located in rural areas and four were located in a mid-size city.

We sampled employees purposively across a range of clinic roles (medical assistants, nurses, physicians, nurse practitioners, social workers, and psychologists) and seniority. We initially reached out to clinic leadership to recruit potential participants, communicated directly with the BH providers, and engaged in a snowball recruitment approach whereby participants invited colleagues to participate. We aimed to enroll approximately 20 participants to reach analytic saturation [35].

### Data collection

Most qualitative semi-structured interviews took place in-person, two were conducted via telephone, and one interview was with two participants. Interviews took place between February 2019 and January 2020. Interviews were conducted by a PhD-trained investigator (CL) and a masters-trained research coordinator (EBN) with three years’ and ten years’ experience in suicide research respectively. Both interviewers identify as female, both have been trained and have experience in qualitative research methods. Four of the interview participants were known professionally to CL before the interview. Participants received a $50 gift card as a token of appreciation. With the goal of implementing the Zero Suicide model in these clinics, the interview topic guide focused on the primary care context, views on several suicide-related practices (including screening and treatment), perceived barriers and facilitators of suicide-related care, and preferred implementation strategies (see Interview Guide, S1 File). Interviews were audio-recorded and transcribed verbatim by an external service compliant with the Health Insurance Portability and Accountability Act. Transcripts were reviewed and checked against audio recordings for accuracy by a researcher (CL) and any identifying information was removed. Recruitment took place between Feb 1 2019 and Jan 22 2020.

### Ethics statement

This study was approved by the UMass Chan Medical School’s Institutional Review Board (IRB) under protocol number H00011407. Using a consent factsheet, participants were informed about the purpose of the study, benefits and risk of participation, the voluntary nature of participation, and compensation. A waiver of written consent was granted by the IRB because participation posed no more than minimal risk to the clinicians and because a signature on an informed consent document would be the only record linking the subject to the research. In a procedure approved by the IRB, participants were presented with the approved consent factsheet and verbal consent was obtained and documented by the researcher before the interview took place.

### Analysis

Transcripts were managed using NVIVO Version 12 software (2018, https://lumivero.com/products/nvivo/). We applied thematic analysis because this is a flexible method that allows both deductive and inductive approaches [36]. We set out to deductively examine the clinical constructs of Identify, Engage, Treat, and Transition from the Zero Suicide model. Although the Zero Suicide model specifies Train, Lead, and Improve as its core implementation strategies, we were interested in a broader set of constructs, namely Context, Implementation Strategies, and Implementation Outcomes, and we applied these as overarching categories along with the clinical constructs listed above. Within these seven categories, we aimed to remain naïve and open to what clinicians felt was most important about these practices. The analytic steps involved immersion/familiarity, generating additional codes from segments of text, collating codes into categories, searching for themes in each category, and reviewing and finalizing themes [36]. CL conducted the initial analyses with assistance from EBN, uncertainties were resolved through weekly discussion, and themes were reviewed and approved by all co-authors. Thematic saturation was achieved.

## Results

We invited a total of 30 clinicians to participate: 4 clinicians did not respond to the invitation, 2 declined, and 24 participated. Our final sample was comprised of seven physicians, four nurse-practitioners, three nurses, four psychologists, five social workers, and one medical assistant (n = 24). Interviews ranged in length from 37 to 79 minutes. Participant characteristics are outlined in Table 1, and themes and subthemes are presented in Table 2.

**Table 1:** Participant characteristics.

Characteristic	n (%)
**Gender**	
** Female**	19 (79%)
** Male**	5 (21%)
**Role**	
** Physician**	7 (29%)
** Nurse**	4 (17%)
** Nurse practitioner**	3 (13%)
** Psychologist**	4 (17%)
** Social worker**	5 (21%)
** Medical assistant**	1 (8%)
**Administrative/leadership role**	
** Yes**	6 (25%)
** No**	18 (75%)
**Age (mean, range)**	46.0 years (24–65)
**Years in clinic (mean, range)**	9.8 years (0.25–36)

**Table 2:** Overview of results and coding scheme.

Theme	Subthemes
1. Context: Busy but committed to our patients	a. Time constraints
	b. Behavioral health (BH) as part of primary care
	c. Teamwork and collegiality d. Longitudinal relationships with patients and families
	e. Acute care as fallback
2. Identification: Diverse ways of assessing (and responding to) suicide risk	a. Different ways of detecting suicidal risk
	b. Varying levels of comfort with suicidality
	c. Assessing social and psychiatric factors
	d. Thresholds of suicidality for decision-making
	e. Assessment is skilled and nuanced
	f. Attending to unspoken communication
	g. Creating psychological safety
	h. Sitting with risk versus escalating
3. Engage: Preserving safety while balancing autonomy	a. Safety planning versus contracting for safety
	b. Planning ahead for a crisis
	c. Assessing lethal means
	d. Least restrictive and shared decision-making
	e. Patient not engaging
	f. Not leaving patient alone
	g. Involving family
4. Treat: Short-term options but long-term shortages	a. Needing a psychiatrist to prescribe medications for serious mental illness (SMI)
	b. Primary care physicians handling medication c. Integrated behavioral health therapy at the clinic (time-limited)
	d. Reimbursement as a challenge
	e. Need for case management/care coordination
5. Transition: We’re not going to let them *not* get cared for”	a. Ability to connect patient to in-house BH
	b. Difficulty connecting to external BH
	c. Emergency Department transition as an (undesirable) option
	d. Acute care shortages
	e. Advocating for the patient
	f. Poor transitions from acute care back to primary care
	g. Communicating about transitions, especially in electronic health record (EHR)
	h. Inappropriate transitions, getting “lumped”
6. Implementation strategies: Support skill-building but don’t overwhelm	a. Developing a clear protocol/policy
	b. Having new assessment and intervention tools
	c. Comprehensive training for all roles
	d. Disseminating information and educational resources
	e. Using continuous quality improvement (CQI)
	f. Changes to the EHR
	g. Improving staffing and shifting roles
7. Implementation outcomes: Placing the patient at the center	a. Patient is satisfied and autonomous
	b. Patient-provider alliance is protected
	c. Patient is safe
	d. Clinician feels confident and supported
	e. Tools and decision-marking are standardized
	f. Flexibility as needed
	g. Clinic flow and care quality is preserved

### Context: Busy but committed to our patients

These integrated primary care clinics were characterized by a fast-moving complex care environment that valued positive, longstanding relationships with patients and colleagues.

Clinics were busy, visits were short and there was a lot of “checking boxes” required by insurers and accreditation organizations, with a “crunch of back-to-back patients” and providers being asked to “do more and more and more”. Clinicians strived to balance clinical quality, efficiency, and patient satisfaction, especially when mental health concerns arose unexpectedly during a visit:

*Sometimes things are dangled… you may not even hear it because you’re busy typing on the computer or you’re interested in filling in a database and they give you a little something and*, *for many reasons*, *time constraints*, *you can say*, *"I don’t want to go down that rabbit hole*. *I’m not ready"* [Physician, Male]

There was variation in willingness for clinicians to have their schedule “thrown off” by delving deeper into patient’s personal issues, with some opting to run behind schedule because “that’s the cost of asking these types of questions”.

The BH needs managed by clinicians spanned a wide range, including depression, anxiety, substance use, post-traumatic stress disorder, and health risk behaviors, as well as more severe mental illness, such as psychosis and bipolar disorder. Embedded BH providers were available to consult with medical providers and for “warm hand-offs”, that is, an immediate introduction to the patient during the medical visit to discuss the identified mental health concern, and shared visits with the patient and physician. Embedded social workers were also available at some clinics to assist with certain tasks, such as addressing housing needs or specialty mental health referrals. Medical providers reported being very satisfied with this integrated BH model of care.

*I set him up with the social worker one day when he was in*. *He was just in a terrible state*. *And it was very helpful*. *She [social worker] saw him like four or five times*. *And then kind of got him through it*. *Gave him some advice*. *And it was hugely helpful*. [Physician, Male]

Patients in a mental health crisis would sometimes show up at the clinic without an appointment, a “crisis is on your doorstep”. When navigating the decision about what to do when mental health emergencies arose, the medical providers appreciated having the opportunity to make a warm hand-off to BH providers. The BH providers in turn sometimes struggled to manage interruptions and “a fair amount of juggling”.

The interviews revealed a strong sense of interdisciplinary collegiality, teamwork, and efficiency within the clinics, with the BH providers abiding by an “open-door policy”:

*You have to be able to bring in people and be able to handle it in real time and have those pieces in place where everyone’s doing their role* [Physician, Male].

A key part of this collegial, integrated approach was the ability to turn to a colleague in the moment, knocking on a door or “catching” them in the hallway, and working as a team with “all-hands-on-deck” in cases of crisis: “this is like a stroke or heart attack, you interrupt whoever you need to interrupt to make sure it’s taken care of" [Social Worker, Female]. Being primary care, these clinics could utilize acute care as a “fallback” if a patient’s mental health situation was more severe.

A final important aspect of the primary care context was long-standing relationships with their patients and their families:

*Primary care providers follow patients across their lifetime*, *they have these longstanding relationships with people*. *They feel comfortable expressing what’s going on*. *They’ll talk about how their kids are*, *what sort of factors are going on in their life*. [Psychologist, Female]

Clinicians were committed to seeing their patients through difficult times. Longitudinal relationships helped providers spot when a patient might be experiencing a downturn in mental health, when “they look more forlorn than I’ve ever seen them”, but the relationship also needed to be preserved carefully, knowing that “if we overreact to the suicidality, it makes the patient less likely to be honest with us next time”.

### Identification: Diverse ways of assessing (and responding to) suicide risk

Clinicians reported that suicidality was detected in a range of ways, sometimes offered by the patient, probed for by the clinician, or detected by screening.

Some patients made unprompted, direct disclosures of suicidality to providers (“It wasn’t like I had to pull it from him”). When patients disclosed their suicidality over the telephone, it led to a flurry of concern as to where the patient was and if they were safe. On occasion, it would be a family member who would raise a concern about the patient, or providers noticed something was off with a patient and created space for disclosure. Clinicians spoke about attending to behavioral cues, both moderating one’s own non-verbal cues and going beyond what was said by the patient to detect risk:

*He said*, *“My toe hurts so bad I could kill myself*,*” and I’m like*, *“You’re saying that in a joking manner*, *but I don’t trust you when you say that in a joking manner”* [Physician, Male].

Similarly, a medical assistant observed that “sometimes it’s not what you say; it’s how you say it too […] body language is a factor, tone is a factor.” Patients who were presenting for a BH reason or who had a history of suicidality were asked about suicidality more routinely:

*I think trying to be open and forthcoming and asking it like another question like*, *“How was your bowel movement*?*” It’s a question that has to be asked for patients*, *especially when there’s history there*. [Physician, Male]

Suicidality was sometimes picked up on routine paper-based depression screening, where some patients “may do better letting you know on paper.”

Once risk was detected, clinicians tended to meet disclosures with understanding, though some of the less experienced staff sometimes “freaked out” or became “dysregulated”:

*They just don’t want to the responsibility because it’s so scary*, *and you’re so alone with it*, *and you can’t avoid it*. [Social Worker, Female].

Clinicians dealing with disclosures by telephone reportedly found them particularly “anxiety-provoking” and “very challenging”.

Once suicidality was detected, the next step was usually for the provider to gather more detail about the patient’s current and past suicidality, as well as psychological, social, and environmental factors, to decide “how serious is this thought” and whether to escalate the patient to a higher level of care. Level of suicidal ideation and past suicidal behavior were key concerns in further assessment by the clinicians. They recognized that patients’ ideation could range from a passive death wish all the way to a detailed high-lethality plan with intent. Medical providers would usually involve integrated BH providers when the patient endorsed either passive or active suicidal ideation, with some physicians tending to “hit the panic button” in response to passive ideation. One BH provider noted that medical providers did not want patients who endorsed suicidality to “sit in their clinic, in their lap and their responsibility.” Besides ideation, the clinicians mentioned other areas they assess, as one provider said, “the more positives, the more questions”. The clinicians often assessed lethal means and whether the plan “had any teeth to it”. Other considerations included history of suicidal behavior, psychiatric history, as well as social (such as recent losses, isolation, financial instability, health issues, suicide by family or friends, bullying), psychological (poor impulse control, hopelessness, perfectionism), and environmental factors (living alone, firearm access, unsafe home environment). Protective factors were less often mentioned but included outpatient treatment engagement, reasons for living, coping strategies, not wanting to hurt friends and family, religiosity, and living with others.

In terms of assessment style, many of the clinicians recognized that a good assessment required openness and comfort on the part of the clinician, which seemed to come with experience. Good assessment also required time and attention, like “a strong, strong connection, [not] staring at a computer screen and checking off boxes while I’m talking to them”.

*I think probably how to toe into it*, *how to move your way into being able to ask a question that’s going to get an authentic response from a patient*, *and to not be afraid of asking the questions that are going to get enough in-depth response* [Nurse Practitioner, Female]

By sitting with risk and assessing thoroughly, clinicians sought to preserve trust and rapport with their patients (“I say like ‘This is a[n] okay place to talk about these things. If you tell me that you want to kill yourself, it doesn’t mean I’m going to send you to the hospital’”). Depending on their training and discipline, “every provider also has a different threshold” in deciding whether to escalate the patient to the emergency department (ED), with some clinicians using the ED as “default”. One provider observed that “there’s no level of comfort with patients with active or even passive SI [suicidal ideation] in our clinic” whereas another said:

*When it’s a vague* [suicide] *plan with no intent*, *I feel very comfortable saying*, *“Okay*, *here’s the safety plan*. *Here’s what the follow up is going to be and we’re going to get you connected*. *We’ll keep a very close eye on you right now but we’re not going to escalate this*.*”* [Social Worker, Female]

A typical escalation to the ED would involve “a person who’s actively suicidal and they have a plan and there’s intent and they won’t contract for safety”, with the threshold for severity seemingly higher among BH providers:

*My threshold for*, *’When do I send somebody to the emergency department*?*" is pretty high*. *[…] I think any sense that they couldn’t make it* [survive] *five to seven days would be*, *"You need to go to the hospital*.*"* [Psychologist, Female]

Clinicians indicated that escalation to acute care shouldn’t be based on a hard cut-off or a number on a screening tool but required the clinician to navigate and weigh complex considerations:

*You would never make a treatment decision based on a single reading and based only on the number*. *You have to place it in clinical context*. *You have to follow up with questions* [Psychologist, Male]

### Engagement: Preserving safety while balancing autonomy

In responding to suicide risk in their patients, clinicians often turned the focus to ensuring the patient’s physical safety in the short term. Although few clinicians described using the full safety planning intervention specifically, a “safety plan” was seen as a useful alternative to the ED for patients who were experiencing active suicidal ideation. BH providers were most likely to mention creating a formal, written safety plan. Some clinicians had more informal safety plans or crisis plans they would create with patients that included coping skills and crisis resources. Planning for a future crisis was something clinicians regularly discussed with patients so “if it goes from ‘I probably won’t’ to ‘I would’, what are you going to do if that sort of change happens?” Clinicians would also try to be upfront with patients about how they would respond if the patient were to deteriorate or stop engaging, with one provider telling patients “this is the date we’re calling. If we can’t reach you, we’re calling for a wellness check [by police]".

The practice of “contracting for safety” was mentioned in multiple interviews. The content of the “contracting” varied but it mostly focused on a verbal or even written agreement to seek help if a suicidal crisis arose. Sometimes, a patient’s willingness or ability to do that was taken as a mitigating factor: “anyone who is a risk when I’m seeing them, who can’t contract for safety, has to be seen and assessed emergently [by BH]”. Several clinicians recognized that the “contract” alone would not keep the patient safe. Many clinicians referred to availability of lethal means, mostly as an assessment rather than as an intervention. Some clinicians mentioned restricting access to lethal means within the clinic itself, such as medical supplies in treatment rooms or, in rural settings, personal firearms:

*I just want to stop and ask*, *“do you have a gun or any other weapon on you right now*?*" and to ask because the other thing*, *we want to be able to tell the police if we’re going to send the police in* [Psychologist, Male]

The few clinicians who mentioned attempting to reduce access to firearms recognized that it was likely to be a sensitive topic:

*It’s not about your right*. *It’s just about your child’s safety*, *and I just want to make sure the gun is locked up secure and they don’t know where it is* [Physician, Male]

Clinicians were mindful about patients being engaged in the decisions around the next steps of care:

W*e do our best to have the conversation and include the patient and help them feel that they’re part of the decision rather than trying to make the decision for them* [Social Worker, Female].

Clinicians generally tried to opt for less restrictive disposition when possible, such as having a next-day appointment or “really close follow up” instead of sending them to the ED. When an ED transfer was needed, some clinicians preferred to have the patient brought there by a family member or friend instead of requiring an ambulance and involuntary commitment to hospital. In letting the patient out of their sight, the provider would seek a commitment that the patient wouldn’t disappear:

*I’m telling a family member*, *"This is on you*. *If they don’t show or if something happens*, *it’s not on me*. [Psychologist, Male].

Clinicians spoke about setting clear expectations so the patient knew what would happen at each step of the care process:

*“I really care about you a lot*, *and I’m really worried about your safety*, *and I think we need to get you more care right now*. *And that’s going to involve going to the emergency room”*, *and then I sort of lay out what that means* [Nurse Practitioner, Female]

The clinicians were keen to avoid involuntarily transferring patients to the ED. “We’re really trying not to section people” as it was “very unpleasant”, “traumatizing”, “slams the door for future work”, and “very painful for a person to have their rights taken away like that”. Ideally, clinicians preferred to have patients engage in care voluntarily, through a process of soft negotiation or a harder line if the patient resisted. Clinicians were unhappy when they had to “resort” to involving police or a section, worried that it could affect patients’ trust and autonomy:

*I have strong ethical feelings about sending somebody under a section*, *somebody who’s willing to go*. *I hate to then take away that autonomy and decision on their part simply because our waiting room is too big or our wait time is too long* [Social Worker, Female]

Part of engaging with the patient was keeping the door open, even “if they’ve no-showed 10 times, if they’ve no-showed 100 times, we are still going to see them”. Family members were also important allies in patient engagement:

*I’d ask the patient*, *"Is it okay if I call your wife*?*" Or if I can’t get in touch with somebody*, *I have called family members just to say*, *"Everything okay*?*" But I don’t do that that often*. *But again*, *I think primary care docs can get away with that a little more comfortably than maybe the gastroenterologist could*. [Physician, Male]

The family could help to keep the patient safe at home, especially in the case of pediatric patients where there was more of an obligation to disclose risk to a caregiver. Occasionally, families could present barriers to engagement, for example when “parents don’t really believe in counseling”, they don’t “go for meds”, or family “culturally not quite understanding the whole concept of mental illness”.

### Treatment: Short-term options but long-term shortages

Social workers and psychologists in these clinics delivered therapeutic interventions at the “intersection of clinical psychology and health psychology”. By design, this integrated BH model is intended to serve patients with lower or emerging acuity with less regularity than what would be seen in mental health outpatient settings, the default appointment being 30 minutes in length. The goal is to deliver lighter-touch therapy across many patients with short wait-times:

*Our behavioral health model is not supposed to be therapy once a week for a year*. *That’s not their role*. *If we really need someone to be in long-term maintenance*, *then we try to find them somebody in the community* [Physician, Male].

BH providers drew on modalities of motivational interviewing, cognitive behavioral therapy, psychoeducation, family systems, cognitive processing, narrative therapy, mindfulness, acceptance and commitment therapy, supportive counseling, and case management. There was little capacity to deliver therapies that target suicidality specifically, like Dialectical Behavior Therapy (DBT) or Collaborative Assessment and Management of Suicidality (CAMS).

Some of the clinics had access to psychiatric consultation on a limited basis, either by a prescriber visiting the clinic weekly or monthly, or through a state psychiatric consultation service for youth. However, several clinics lacked a formal consultation link with psychiatry and faced months-long wait times for referrals, with one clinician observing that “No clinic is going to tell you they have enough psychiatric support”. The primary care prescribers felt comfortable prescribing common medications like antidepressants or renewing less common medications for serious mental illness, especially in collaboration with a psychiatrist:

*I’ve renewed those* [schizophrenia] *medications and*, *as long as they’re stable*, *I do that*. *But if there’s changes*, *I depend on the psychiatrist to help with that* [Physician, Male]

Providers felt “some of the antipsychotic medications get a little tricky” for primary care providers to manage alone: “initiating somebody who’s never been on these like antipsychotics or mood stabilizers, they’re less comfortable and I don’t think they should be, they’re family medicine doctors” and “there can be times where it feels like we’re sort of managing [medication] on our own, but we really shouldn’t be”. With particularly challenging cases, the dearth of specialty care gave clinicians a sense of abandonment by the system, with one social worker saying "We need help. Someone, send us that life raft because we need help”.

Reimbursement for mental health treatment was a recurring issue affecting treatment options, especially within an integrated care model. Some providers mentioned not being able to bill for mental health work done, others were able to bill for a crisis code if it exceeded the therapy code time. There were challenges to referring patients to specialty mental health because of external providers not accepting their insurance. Several providers spoke of frustrations with how mental health care is generally valued and the lack of parity with physical health care, with a nurse observing that “there isn’t equality between physical and mental health…It’s terrible. It’s just not valued yet.”

### Transition: “We’re not going to let them *not* get cared for”

There were several types of transitions happening for patients with suicide risk, including in-house BH, specialty outpatient psychiatric care, and acute care.

Medical providers in the clinics we examined had the option to connect patients experiencing suicidal crisis to in-house BH providers for short-term intervention. That warm hand-off needed to be timely to ensure safety. A BH provider was then often able to make a timely assessment and advise whether the patient needed acute care or a BH follow-up appointment. Making warm hand-offs to in-house BH providers was seen as a good route to behavioral healthcare, allowing for more thorough assessment and leading to higher retention.

*It’s less threatening to say*, *"I’m going to walk down the hall*,*" as opposed to*, *"I want you to call up and get an appointment*.*" It’s really lowered the bar for getting some people in* [Psychologist, Male]

Though integrated BH was highly valued within the clinics, staffing was somewhat sparse, with periods of time when there were no BH providers available for a warm hand-off or rapid follow-up.

For more complex cases, providers attempted to link patients to specialty outpatient BH care for longer, more frequent appointments focusing on more severe mental health concerns. This process was described by several clinicians as being very difficult. Clinicians viewed the system as “fragmented”, with limited capacity, financial concerns, and patchy insurance coverage, that were getting worse over time:

*In the community*, *it’s just become harder and harder*. *It’s very hard to refer to an individual*. *And I’m just sad for the patients because they’re really struggling* [Physician, Male]

It was “not an easy process” for patients to navigate. When patients did secure a specialty outpatient BH care provider, the primary care clinics had varied success in obtaining updates and collaborating with them. There were also several patient-side barriers reported by clinicians, including transportation challenges, personal organization, ambivalence, and a reluctance to engage.

If clinicians detected imminent risk during a clinic visit, they would consider sending the patient to the ED and in that transition, patient safety and buy-in were key concerns. When needed, clinicians tried to help to smooth the transition to the ED by advocating on behalf of the patient to minimize wait times and avoid the patient “fall[ing] through the cracks” by not being admitted. One psychologist commented that “if I really want someone to get a bed, I tend to write a note that makes it very clear why I sent them and hope that gets them over the line”. Some clinicians located close to an ED physically accompanied patients there to ensure a safe transition and “to shortcut the whole procedure”.

It was frustrating for clinicians when a patient they were sure was at imminent risk was discharged from the ED and not hospitalized. One physician noted that sometimes he didn’t “think that going to the ED is going to help this person. There’s no way they’re going to admit him. It’s just going to ruin their week. But do we have to do it anyways?". This subtheme came up several times:

*If you section somebody*, *they have to sit in the ED for hours before they can get assessed*. *They get assessed and then there’s no help for them*. *Why are we going to do that to them*? *They’re going to distrust me now even as much as they distrust the ED*. [Social Worker, Female]

The ED itself was described as being “overcrowded”, “unwelcoming”, and “difficult”, and several clinicians felt that EDs were under severe pressure and rarely contacted the clinic for collateral information or to give an update. Clinicians mentioned desired alternatives to the ED, like community-based mobile crisis and crisis intervention units.

Conversely, patients were often discharged from acute care back to primary care. Some clinicians were frustrated by the tendency to discharge a patient from acute care without engaging primary care, establishing a psychiatrist for them, or sharing notes from the hospitalization:

*We don’t know what they received while they were there or anything like that*. *So it makes it really hard*. *And we’re asking the patient who may or may not recall the whole hospitalization or depending on the experience—so that’s very*, *very challenging* [Social Worker, Female]

Clinicians mentioned that note-sharing from inpatient care may have been restricted because of confidentiality concerns. It was up to the clinician to “chase them down to give me discharge notes or a plan”. Other clinicians had more positive experiences of being able to access inpatient notes and being consulted by providers during their patient’s admission:

*They’re reaching out to me and just sort of getting what I heard*, *what I saw*, *what I did on my end*. *And then also talking about the treatment plan and giving me a sense of what their next steps are going to be* [Social Worker, Female]

Despite their frustrations, clinicians tried their best to secure good transitions for their patients among levels of care:

*I feel like if they’re not an imminent threat and sectionable that pretty much we’re going to end up taking care of them and that’s not really our role*. *But we’re not going to let them not get cared for*. [Psychologist, Female]

Generally, the primary care clinicians felt that transitions within the same clinic went smoothly. However, transitions to and from acute care, and to specialty outpatient care were characterized by poor communication and a dearth of resources and support. This led to a perception of poorer patient outcomes and clinician frustration, especially when they felt that they were the only person taking responsibility for the patient’s wellbeing.

### Implementation strategies: Support skill-building but don’t overwhelm

In preparation for implementing the Zero Suicide model in these clinics, we sought clinicians’ perspectives on appropriate implementation strategies for the setting. Many clinicians expressed the need for clearer protocols around suicide risk management, so that clinicians would “at least know what to do next”. Nurses and medical assistants in particular preferred guidance to be quite structured, like a “decision tree” or “an exact play by play”:

*It makes you feel better to know that there’s going to be something so you know what to do exactly or steps to take*. *So I think it’s very important to have that* [Medical assistant, Female]

Several clinicians felt that protocols for providers shouldn’t be “black and white” but that having some guidance would help to avoid unnecessary patient restrictions, mitigate risk, alleviate worry, and manage limited resources. Clinicians were open to using suicide screening and assessment tools, especially if they were “shorter and quicker and easier” and “very minimal distraction from the face-to-face encounter”:

*I think from a liability standpoint it’s probably a good idea to have*, *right*. *You can see that structure to make sure we’re not missing important questions* [Psychologist, Female].

Clinicians also pointed to the need for training, ideally being “interactive” and “hands-on”. There was a sense that suicide risk management was not taught consistently in clinical education programs, and that every clinical role would benefit from more training in this topic:

*Especially with the front desk and the triage folks*, *if someone’s calling in*, *they get those first*. *And so how do you manage that*? *Who do you reach out to*? *And how do you sort of keep calm yourself and make sure the patient is calm*? *Or if someone walks in off the street*, *that also happens* [Social worker, Female]

Learning happened informally in the clinics as well, where newer colleagues learned from more established ones and different disciplines learned from one another. Several participants mentioned the need for enduring learning resources around suicide, like videos, instruction documents, and other products that would be available “just in time.” It was noted that the emergent nature of a crisis could make it difficult to be able to use these tools in real time:

*It’s really just figuring out in that moment*. *Like you can throw me all the tools and screenings and questions*. *But I don’t know if I’d use them at that point* [Social Worker, Female].

Participants recommended repeating messaging across multiple modalities and types of meetings and suggested several continuous quality improvement-type strategies, such as sharing practice-level data, using idea boards, convening huddles, reviewing adverse events, and piloting new processes.

Clinicians suggested that the electronic health record (EHR) could be helpful in managing patients experiencing suicidal crisis by adding, for example, longitudinal visibility of risk information, secure patient messaging, telehealth, reminders to screen, and smart phrases. There were several EHR-related concerns about the added burden of documentation, such as “alarm fatigue”, “more clicks”, the inadequacy of “boilerplate language”, and the fear that “the computer is a huge barrier to authentic connection with patients”.

Many clinicians felt that staffing changes would help. For example, they felt that medical assistants could help with mental health screening and scribes could help with patient observation. Many of the clinics were in need of additional social worker and psychologist capacity. Some clinicians pointed to issues around scope of practice in certain roles:

*I think [having medical assistants to screen for suicide risk] puts them in a really hard place*. *I just don’t think that’s fair to them*. *I don’t think it’s fair maybe from a liability standpoint but more so*, *I’m worried about that individual and what they’re taking on* [Psychologist, Female]

As well as practical implementation strategies, participants described strategies to increase buy-in within the clinics. These suggestions included focusing implementation messaging on more “sympathetic” populations of patients (such as children), seeking consensus among teams, and obtaining leadership endorsement.

### Implementation outcomes: Placing the patient at the center

Clinicians desired several outcomes for their patients: that the patient would be satisfied with their care, not distressed, or unduly restricted, safe, and on the road to recovery. Clinicians wanted patients to feel “like someone is caring on the other end” and were keenly aware that patients experiencing suicidal crisis regularly had negative experiences in acute care. Several clinicians shared stories about being there for their patient in dire circumstances, for example, staying in the ED with a sectioned primary care patient who was “openly weeping”:

*And I just stayed with her*, *like*, *"This is the right thing to do*. *This is what you need right now*. *I know it’s not ideal"*, *just locking eyes with her*, *and “just trust me*. *This is where we need to be*. [Nurse-practitioner, Female]

Clinicians often had to balance a concern about patient safety and potential liability with their desire to protect patients’ autonomy, dignity, and psychological safety:

*We know that the pure risk management approach would always be to call the ambulance and have them go*, *and then we would have no exposure then once we hand them off*. *But it’s a traumatizing experience* [Psychologist, Male].

Clinicians sought to preserve their relationship and rapport with the patient, even amid a safety crisis, “just trying to keep that relationship with the individual”. They described witnessing patients’ recovery in the longer term, with several clinicians describing patients who had survived a suicide attempt as now “thriving” and “flourishing”.

Clinicians desired several outcomes for themselves. They wanted to feel confident, autonomous, and supported in addressing suicide risk. They spoke of feeling or wanting to feel “comfortable” and “confident”, often by having a better idea of how to respond to patients experiencing suicidal crisis. Overall, the clinicians suggested that knowing more about how to manage suicide risk would help them “feel better”. They expressed a wish for “clear workflow for everyone as well so that it’s not so chaotic” and more standardization of processes around suicide-related care:

*If there was standard work for suicidal patients*, *then really anybody in our clinic could keep them safe while the behavioral health team does what they need to do* [Social worker, Female]

Any standardization, however, needed to have flexibility for the clinicians to use their clinical judgment as there was a sense that overly restrictive policies could lead to worse care, for example “you would never make a treatment decision based on a single reading and based only on the number”. Finally, clinicians wanted BH care to be timely, high-quality, and accessible for primary care patients who needed it.

## Discussion

In this study, we conducted qualitative interviews to explore clinicians’ perspectives on identifying, engaging, treating, and transitioning patients with suicide risk in the integrated primary care setting. It was clear that these clinics were very busy and fast-paced but valued their long-standing relationships with patients and families. Suicide risk was identified in a wide variety of ways, and clinicians sought to understand more about a patient’s risk before acting. The clinicians had many of the skills required to engage and treat patients with suicide risk but expressed interest in further training, tools, and support in these endeavors. Our participants had some familiarity with safety planning, a brief intervention for engaging patients with suicide risk but also spoke regularly of “contracting for safety”. Providers were generally comfortable in prescribing antidepressants but less comfortable with more complex medications for serious mental illness. Clinicians wanted patients to be both physically and psychologically safe, and to be able to transition to specialty treatment as needed. Clinicians in our study felt that transitioning patients to acute care ought to be a last resort because that transfer had the potential to negatively impact the patient and clinicians’ rapport with the patient. Similarly, the discharge from acute care back to primary care was also fraught, characterized by a lack of communication and planning Internal challenges included juggling competing demands, uncertainties in how to respond to suicide risk, concerns around safety and liability, and a wish to preserve their relationship with the patient. Externally, challenges included shortages in specialized care, suboptimal transitions with acute care, and broader concerns about how mental health was valued and prioritized in the health care system.

One way to improve suicide risk detection is through universal suicide screening, which is recommended by the Joint Commission in acute care settings under the National Patient Safety Goal (NPSG) 15.01.01 and may be feasible and effective in primary care [37–39]. Several suicide risk screening tools have been developed for the acute care setting, but it remains to be seen how they would operate in the primary care setting. Previous qualitative studies of primary care providers [9,23] have echoed concerns about what to do once suicide risk is detected. Safety planning, a brief intervention for engaging patients with suicide risk that has substantial empirical support [12] should not be conflated with “contracting for safety”, an outdated practice now seen as potentially harmful and counterproductive [40,41]. MacDonald et al.’s systematic review [42] that patients presenting with suicidality report experiencing care that “ranges from gentle to hostile”. From a patient perspective, “safety” in the inpatient setting includes feeling psychologically safe by forging connection with providers and being able to re-establish a sense of control [43]. Providers in our study were comfortable in prescribing antidepressants, which appear to be effective in preventing suicide attempts [44]. Like almost half of the US population, our participants live in a mental health workforce shortage area [45], with current psychiatrist staffing in the state meeting only one-third of the actual need. Several large-scale studies [46,47] found that most primary care physicians do not have the experience needed to manage complex psychotropic regimens without psychiatrist support. Discharge planning from psychiatric inpatient care should include communicating with an outpatient provider prior to discharge, scheduling an aftercare appointment, and forwarding a discharge summary [48]. The period immediately following discharge from psychiatric hospitalization is high-risk for suicide [49] and Riblet et al. [50] found that poor communication around suicide risk was a significant root cause in a series of suicides during that transitionary period. Primary care teams can be an important safety net during the transition from acute care back to the community, but they are often overlooked, with existing guidelines omitting primary care as a partner in that transition. The gaps in training and competencies around suicide risk management we observed have also been noted in several other studies [20,38,51].

Overall, our findings suggest that a Zero Suicide type model could fit well in a primary care setting that has integrated BH; our participants were keen to improve processes and skills around identification, engagement, treatment, and transition of patients with suicide risk. Many clinics routinely use the Patient Health Questionnaire-9 [52], which includes an item about passive and active suicidal ideation. A positive value on that question could be followed up with a dedicated suicide screening tool. Empirically derived tools with predictive validity include the Columbia-Suicide Severity Rating Scale screening version [53], Ask Suicide-Screening Questions [ASQ] screener [54], and the Patient Safety Screener [55]. Such screeners should be followed by a more in-depth psychosocial assessment as needed. If imminent risk to life is detected, the patient may require transition to a higher level of care, ideally emergency mental health services or mobile crisis services. If non-imminent risk is detected, the patient could benefit from brief counseling, referral to BH, safety planning, lethal means counseling and a medication review. It would be important to ensure that such an approach would be implemented in a flexible way that prioritizes the relationship between provider and patient, respects the autonomy of both patient and clinician, and adapts to the unique resources and strengths of each clinic.

### Limitations and strengths

The current study had several limitations. First, the setting was primary care clinics with an integrated model of BH and embedded BH clinicians; it is not clear how these findings might apply to primary care clinics without any BH providers. It is reasonable to assume that primary care clinics without integrated BH teams would feel even less equipped to assess and manage acutely suicidal patients. Second, these interviews took place before the COVID-19 pandemic, and it is unclear whether or how the pandemic has impacted these findings. Finally, although we attempted to recruit a wide range of clinical staff from primary care, we did not have sufficient representation from medical assistants. Nonetheless, we believe this is the first study to examine clinician perspectives on applying the Zero Suicide model in primary care. This study is also strengthened by its representation of clinicians from a variety of disciplines.

## Conclusion

This qualitative study demonstrated that current suicide-related practice at participating clinics reflected many of the components of the Zero Suicide model and unearthed many challenges faced by primary care clinicians in caring for patients with suicide risk. While a clinic may be able to improve processes of suicide risk detection and management internally, these efforts will be stymied unless suicide-related care is a priority across the whole healthcare system. Future research should address the feasibility of the Zero Suicide model in primary care settings that do not have integrated BH, particularly around the viability of brief interventions and transitions for patients experiencing suicidality in those settings.

## Supporting information

S1 File Qualitative interview guide. (DOCX)
