# Supplementary material for: “It’s so scary, and you’re so alone with it”: Clinicians’ perspectives on suicide risk management in integrated primary care
Source: PLOS Ment Health. 2024 Jun 4;1(1):e0000029. doi: 10.1371/journal.pmen.0000029 (PMC12798596; doi:10.1371/journal.pmen.0000029)
Supplement: S1 File — (DOCX) [file pmen.0000029.s001.docx]

**Supplemental File: Interview Guide**

1. **Mental health care in the clinic– 10 minutes “I am interested in how you currently provide mental health care in the clinic. Can you tell me about that?”**

**Probes:**

- What kinds of mental health problems do you encounter in your patients?
- Can you describe a recent case? E.g. depression, serious mental illness
- How are mental health problems usually identified in patients (screening, patient complaint, communication from other care settings)? How are they managed?
- How do you feel about managing mental health concerns in this setting?

1. **Suicide care in the clinic– 15 minutes “What about suicide prevention and care here in your clinic? What are you doing now?”**

**Probes:**

- Have you ever encountered suicide risk in a patient in the clinic? Can you please tell me the story of how you found out and what happened? How did you feel about that?
- Are there any standard clinic protocols that must be followed to identify and manage suicide risk? Any tools, trainings, key people or partnerships that are available?
- What do you wish you had at the time?
- Were changes made to protocols or practice as a result of this experience? Please describe these changes. Did these changes help?

1. **Suicide prevention program- 15 minutes “I am interested in your thoughts about the ideal suicide prevention program in a clinic like yours… what would that be like?”**

**Probes:**

- If you were designing a suicide prevention program for this clinic, what would that look like?
  - - How would you identify risk? Would you do screening, with whom? Why?
    - What would you do with those identified as having risk?
      - Assessment, safety, treatment, follow-up?
  - Implementation
    - How would you implement these practices? (e.g. training, EHR, resources, protocols)
    - What do you think it would take to keep that new program going once it’s implemented?
  - What do you think of this example protocol for primary care (IFH)?
  - What do you think of this protocol we implemented in the ED? What would look different in primary care?
